# The causal association between inflammatory bowel disease and breast cancer: a bidirectional two-sample Mendelian randomization study

**DOI:** 10.3389/fgene.2024.1392341

**Published:** 2024-07-23

**Authors:** Yulai Yin, Xiaoyu Zhang

**Affiliations:** ^1^ Cangzhou Central Hospital, Hebei Medical University, Cangzhou, China; ^2^ Department of Thyroid and Breast Surgery Ⅲ, Cangzhou Central Hospital, Cangzhou, China

**Keywords:** inflammatory bowel disease, Crohn’s disease, ulcerative colitis, breast cancer, Mendelian randomization

## Abstract

**Objective:** This Mendelian Randomization (MR) study aims to explore the potential bidirectional causal relationship between Inflammatory Bowel Disease (IBD) and Breast Cancer (BC).

**Materials and Methods:** We utilized genetic instruments from the summary statistics of genome-wide association studies (GWAS) on IBD among individuals of European ancestry (12,882 cases and 21,770 controls) to investigate the association with breast cancer (14,910 cases and 17,588 controls) and *vice versa*. The primary causal estimates were obtained using the Inverse Variance Weighting Method (IVW), and the robustness of the results was evaluated through a series of sensitivity analyses.

**Results:** The study found a positive impact of genetically predicted IBD on breast cancer (OR = 1.047; 95% CI:1.009–1.087; *p* = 0.014); in the analysis of IBD subtypes, genetically predicted Crohn’s Disease (CD) also had a positive effect on breast cancer (OR = 1.044; 95% CI:1.015–1.073; *p* = 0.002), but genetically predicted Ulcerative Colitis (UC) did not show a significant effect on breast cancer (*p* > 0.05). The reverse Mendelian Randomization analysis indicated that genetically predicted breast cancer promoted the overall occurrence of IBD (OR = 1.112; 95% CI:1.022–1.211; *p* = 0.014); however, genetically predicted breast cancer did not show a significant correlation with IBD subtypes (CD and UC) (*p* > 0.05). Genetic predictions indicate a positive effect of Crohn’s Disease (CD) on the risk of Estrogen Receptor-Positive Breast Cancer (ER + BC), with (OR = 1.021; 95% CI:1.002–1.040; *p* = 0.002). Furthermore, a reverse Mendelian randomization analysis reveals that genetically predicted ER + BC contributes to the increased incidence of ulcerative colitis (UC), as indicated by (OR = 1.098; 95% CI:1.032–1.168; *p* = 0.003). In contrast, genetically predicted Estrogen Receptor-Negative Breast Cancer (ER-BC) has been shown to promote the overall occurrence of inflammatory bowel disease (IBD), with (OR = 1.153; 95% CI:1.008–1.319; *p* = 0.037). However, bidirectional two-sample Mendelian randomization analyses between other pairs did not reveal any significant associations (*p* > 0.05).

**Conclusion:** This study elucidates the bidirectional causal association between breast cancer and inflammatory bowel disease, highlighting the necessity of screening for IBD in breast cancer patients and for breast cancer in IBD patients in clinical settings.

## Introduction

Inflammatory Bowel Disease (IBD) (El Hadad et al., 2024; Ghouri et al., 2020; Hemmer et al., 2023; M’koma, 2022) represents a group of chronic intestinal inflammatory conditions characterized by persistent and relapsing clinical features. It primarily encompasses two subtypes: Crohn’s Disease (CD) and Ulcerative Colitis (UC). IBD often presents with significant intestinal and extraintestinal symptoms, though there is variability among individuals. Due to the chronic and recurrent nature of IBD, as well as its associated risk of colorectal cancer transformation (Gao et al., 2023), patients often experience varying degrees of psychological distress. Breast cancer is a malignant tumor originating in breast tissue (Britt et al., 2020; Tsang and Tse, 2020; Derakhshan and Reis-Filho, 2022; Katsura et al., 2022), and it is the most common malignancy among women, with rare occurrences in men. Symptoms leading to clinical presentation often include breast lumps, skin indentations, “orange peel” texture changes, and enlarged axillary lymph nodes. Some patients in advanced stages may exhibit skin ulceration and secondary symptoms due to tumor metastasis beyond the breast tissue. Based on the expression status of Human Epidermal Growth Factor Receptor 2 (HER2), Estrogen Receptor (ER), and Progesterone Receptor (PR), breast cancer can be classified into four molecular subtypes: Luminal A, Luminal B, HER2-positive, and triple-negative breast cancer. Currently, the causal association between IBD and breast cancer remains contentious, with a lack of large cohort studies or randomized controlled trials to elucidate their causal relationship.

Mendelian Randomization (MR) studies (Bowden and Holmes, 2019; Carter et al., 2021; Birney, 2022; Bonilla et al., 2023) primarily rely on single nucleotide polymorphisms (SNPs) to infer the causal relationship between exposures and disease outcomes through genetic variations. In MR studies, phenotype-associated genetic variations are used as instrumental variables for exposures, enabling causal inference of exposure-outcome associations. Genetic variations follow the principle of alleles being randomly segregated from parents to offspring and are determined by genetic variants at conception, thereby minimizing the impact of confounding factors common in traditional observational studies. To elucidate the causal associations between inflammatory bowel disease (IBD) and its subtypes—Crohn’s disease (CD) and ulcerative colitis (UC)—and breast cancer along with its subtypes—Estrogen Receptor-Positive (ER + BC) and Estrogen Receptor-Negative Breast Cancer (ER-BC), this study employs bidirectional two-sample Mendelian randomization analysis. This approach facilitates an investigation into the genetic interrelations and potential causative pathways linking these prevalent diseases and their variants.

## Materials and methods

### Selection of instrumental variables

For the Mendelian Randomization (MR) study to yield valid and reliable conclusions, the selection of instrumental variables (IVs) must satisfy three assumptions:

1) Genetic variations must be strongly associated with the exposure.
2) The genetic variations must influence the outcome solely through the exposure, not directly or through other pathways.
3) The genetic variations must not be associated with any confounders of the exposure-outcome relationship.

For the study of breast cancer and its subtypes, this investigation utilized summary statistics from genome-wide association studies (GWAS) conducted by the Breast Cancer Association Consortium. The analysis included data on breast cancer overall (BC) comprising 14,910 cases and 17,588 European ancestry controls, Estrogen Receptor-Positive Breast Cancer (ER + BC) with 69,501 cases and 105,974 controls, and Estrogen Receptor-Negative Breast Cancer (ER-BC) involving 21,468 cases and the same 105,974 controls of European descent. Regarding inflammatory bowel disease (IBD), summary statistics from the GWAS by the International Genetics of IBD Consortium included data on European participants, covering the entire spectrum of IBD (12,882 cases and 21,770 controls), as well as the specific disease entities Crohn’s disease (CD) with 5,956 cases and 14,927 controls, and ulcerative colitis (UC) with 5,371 cases and 412,561 controls.

### Minimizing the effects of linkage disequilibrium

To minimize the effects of Linkage Disequilibrium (LD) (Meisinger and Freuer, 2022; Wang et al., 2023), we selected Single Nucleotide Polymorphisms (SNPs) as IVs that passed the recognized genome-wide significance threshold (*p* < 5 × 10 ^-8, r^2 ≤ 0.001, meeting Hardy-Weinberg equilibrium (H-W), and genetic distance <10000 kb). The F-value of IVs was calculated to ensure the inclusion of IVs with F > 10, thereby avoiding bias from weak instrumental variables. To facilitate the forward Mendelian randomization analyses, wherein inflammatory bowel disease (IBD), Crohn’s disease (CD), and ulcerative colitis (UC) serve as exposures with breast cancer and its subtypes as outcomes, we selected 130, 53, and 21 single nucleotide polymorphisms (SNPs) as instrumental variables, respectively. For the reverse Mendelian randomization analyses, where breast cancer and its subtypes (ER + BC and ER-BC) are considered exposures, we employed 17, 106, and 37 SNPs related to these exposures as instrumental variables, respectively. This strategic use of genetic instruments aims to elucidate potential causal relationships between these complex diseases.

### Data availability

Detailed summary data for the genome-wide association studies (GWAS) of breast cancer and its subtypes are provided in Supplementary Material S1. Correspondingly, summary statistics related to GWAS for inflammatory bowel disease (IBD), Crohn’s disease (CD), and ulcerative colitis (UC) can be found in Supplementary Material S2. These documents contain comprehensive genetic insights critical for the analyses presented in this study.

### Statistical analysis

The primary method for our analysis was the Inverse Variance Weighted (IVW) Mendelian Randomization (MR) approach. We assessed heterogeneity using the IVW and MR-Egger methods, applying Cochran Q and Rücker Q tests. Multiple testing for pleiotropy was conducted using the Egger-intercept method, and sensitivity analyses were performed through the leave-one-out approach.

To bolster the evidence, an independent MR study was conducted using the PhenoScanner database to exclude pleiotropic SNPs associated with confounders of the exposure-outcome relationship.

Effect estimates are presented as Odds Ratios (ORs) with 95% Confidence Intervals (CIs).

For subtypes of IBD, the genetic correlation between CD and UC was estimated by calculating the correlation of effect sizes on a logarithmic scale (i.e., β).

Due to multiple testing considerations, associations with *p* values less than the Bonferroni correction threshold of α = 0.05/18 = 0.0028 are considered statistically significant. Meanwhile, associations with *p* values ≥0.0028 and <0.05 are regarded as suggestive of significance. This approach ensures rigorous evaluation of the statistical evidence supporting the associations under study. All analyses were conducted using the open-source statistical software R (version: 4.3.2). The reporting adheres to the STROBE-MR (Skrivankova et al., 2021a; Skrivankova et al., 2021b) (Strengthening the Reporting of Observational Studies in Epidemiology-Mendelian Randomization) statement.

### Ethical statement

This study was conducted based on publicly available Genome-Wide Association Studies (GWAS) databases. All the original studies had received ethical approval.

## Results

In the subtypes of Inflammatory Bowel Disease (IBD), Crohn’s Disease (CD) and Ulcerative Colitis (UC) were found to have a weak genetic correlation, suggesting that these subtypes are not likely to occur systematically together.

**FIGURE 1:**
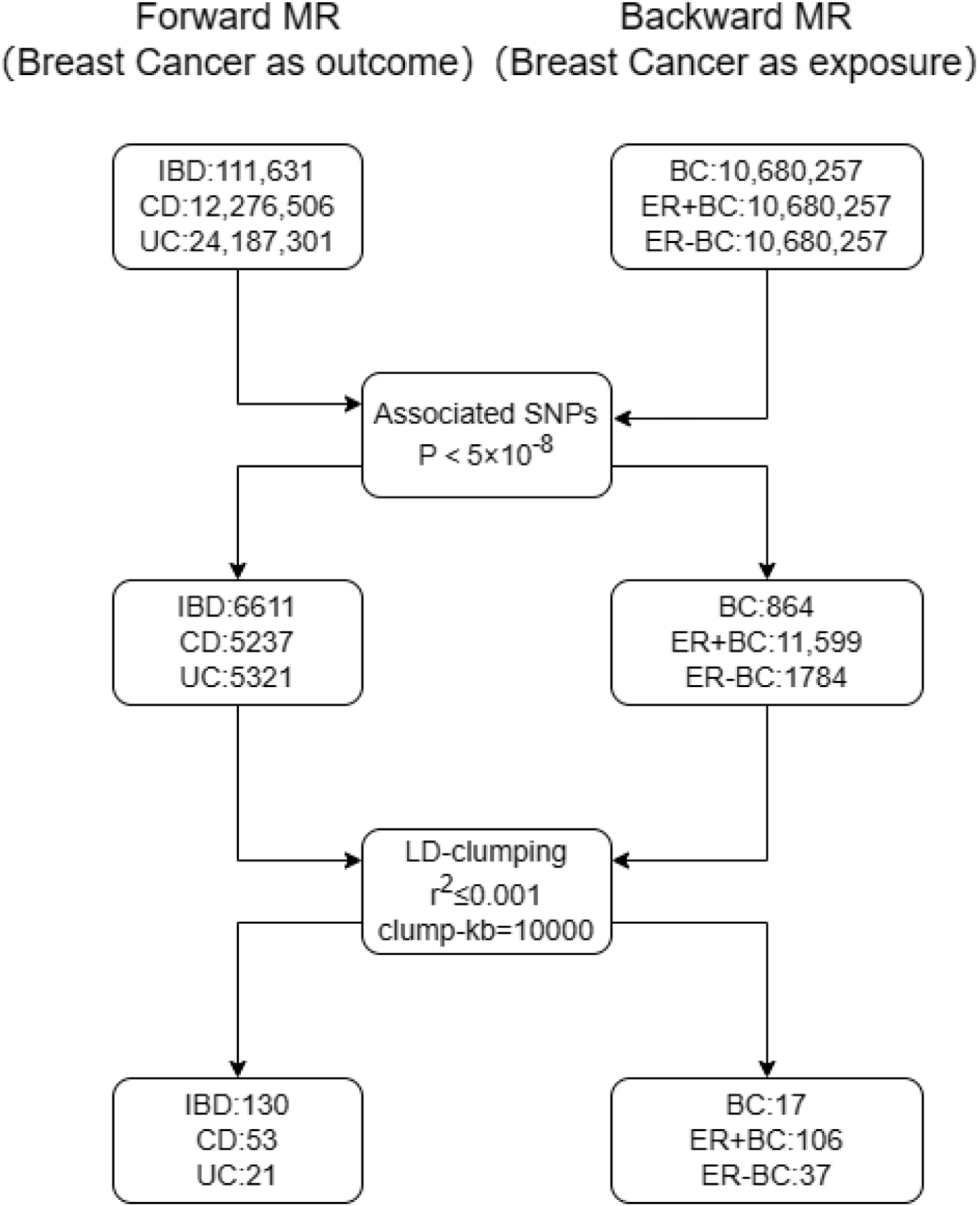
The SNP selection processes for Mendelian randomization analyses are depicted. The left panel details the steps for selecting instrumental variables for forward Mendelian randomization with breast cancer and its subtypes as outcomes. The first step involves excluding SNPs with association *p*-values ≥5 × 10 ^ -8, and the second step excludes SNPs with linkage disequilibrium (r ^ 2 > 0.001) or proximity within 10,000 kilobases. The right panel illustrates the SNP selection process for reverse Mendelian randomization, where breast cancer and its subtypes act as exposures.

**FIGURE 2:**
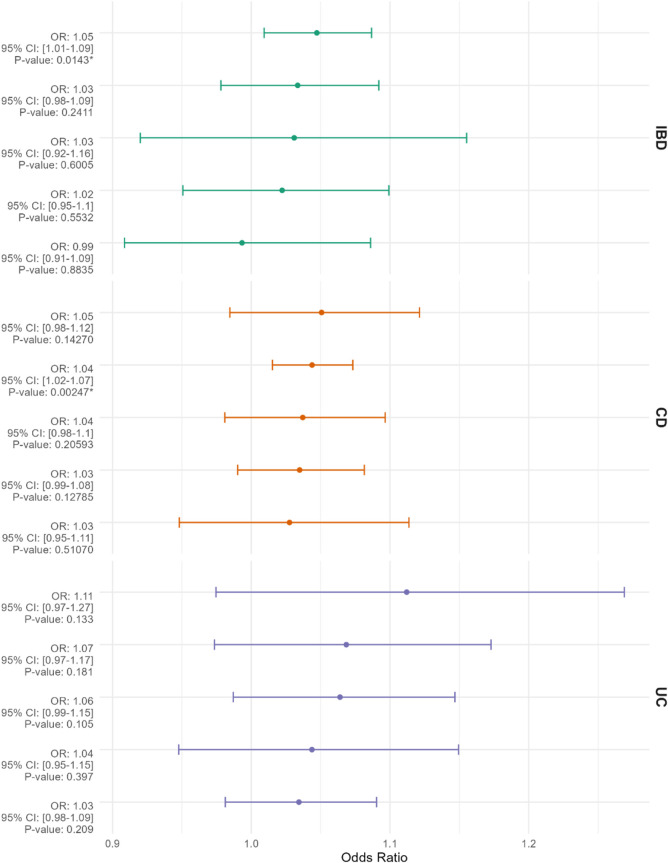
The Mendelian randomization results, displayed from top to bottom, evaluate inflammatory bowel disease (IBD), Crohn’s disease (CD), and ulcerative colitis (UC) as exposures with breast cancer (BC) as the outcome, presented as odds ratios (ORs) with 95% confidence intervals. The methods employed in the Mendelian randomization analysis include “MR Egger,” “Weighted Median,” “Inverse Variance Weighted,” “Simple Mode,” and “Weighted Mode,” arranged from top to bottom based on descending OR values. An asterisk (*) denotes *p* < 0.05.

**FIGURE 3:**
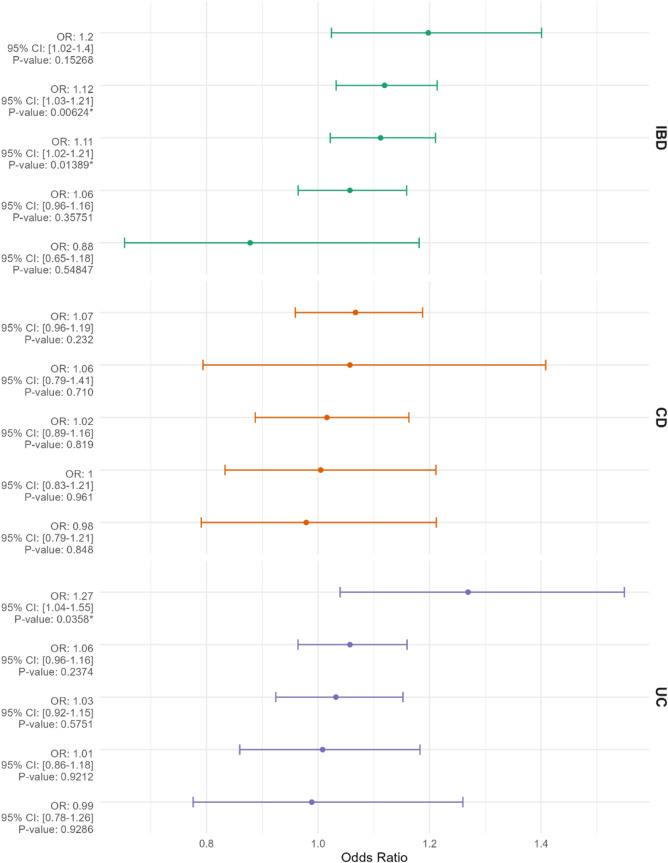
The Mendelian randomization results presented from top to bottom assess breast cancer (BC) as the exposure and inflammatory bowel disease (IBD), Crohn’s disease (CD), and ulcerative colitis (UC) as the outcomes, quantified as odds ratios (ORs) with 95% confidence intervals. The methods utilized in the Mendelian randomization analysis include “MR Egger,” “Weighted Median,” “Inverse Variance Weighted,” “Simple Mode,” and “Weighted Mode,” organized in descending order of OR values from top to bottom. An asterisk (*) signifies *p* < 0.05.

The selection process for Single Nucleotide Polymorphisms (SNPs) is depicted in Figure 1. Utilizing the Inverse Variance Weighted (IVW) method as the primary analytical approach, the estimated values are reported as follows. A positive correlation was observed between genetically predicted Inflammatory Bowel Disease (IBD) and Breast Cancer (BC), with an Odds Ratio (OR) of 1.047; 95% Confidence Interval (CI): 1.009–1.087; *p* = 0.014 (Figure 2). In the analysis of two subtypes of IBD, Crohn’s Disease (CD) showed a positive correlation with BC (OR = 1.044; 95%CI:1.015–1.073; *p* = 0.002); however, Ulcerative Colitis (UC) exhibited no significant correlation with BC (Figure 2). Reverse Mendelian Randomization analysis indicated a positive correlation between genetically predicted BC and IBD (OR = 1.112; 95%CI:1.022–1.211; *p* = 0.014) (Figure 3), with no significant correlation found between BC and either CD or UC (Figure 3).

**FIGURE 4:**
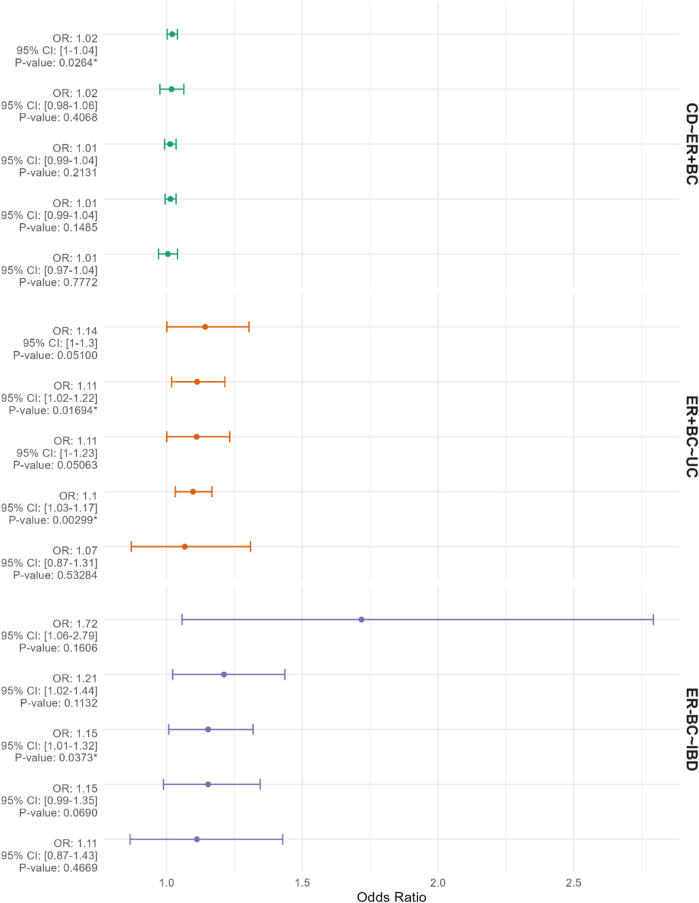
The figure presents Mendelian randomization results for corresponding exposures and outcomes from top to bottom. The factors listed before the “∼” represent the exposures, while those listed after the “∼” represent the outcomes. The results are depicted using odds ratios (OR) and 95% confidence intervals (CIs). The methods employed for the Mendelian randomization analyses include “MR Egger,” “Weighted median,” “Inverse variance weighted,” “Simple mode,” and “Weighted mode.” The results are arranged in descending order of OR values. An asterisk (*) indicates *p* < 0.05.

In the context of breast cancer subtypes, genetic prediction of CD demonstrates a positive effect on estrogen receptor-positive breast cancer (ER + BC) (OR = 1.021; 95% CI: 1.002-1.040; *p* = 0.002) (Figure 4). Conversely, reverse Mendelian randomization analysis reveals that genetically predicted estrogen receptor-positive breast cancer increases the incidence of UC (OR = 1.098; 95% CI: 1.032-1.168; *p* = 0.003) (Figure 4). Furthermore, genetically predicted estrogen receptor-negative breast cancer (ER-BC) is associated with an elevated risk of IBD overall (OR = 1.153; 95% CI: 1.008-1.319; *p* = 0.037) (Figure 4). No significant associations were observed between other pairs in the two-sample Mendelian randomization analysis (*p* > 0.05). Heterogeneity tests, including Cochran’s Q and Rücker’s Q using IVW and MR-Egger methods, are detailed in Supplementary File S3. All Mendelian randomization analyses employed the Egger-intercept method for pleiotropy assessment, which did not indicate any pleiotropy (*p* > 0.05), as presented in Supplementary File S4.

## Discussion

This study found a positive correlation between genetically predicted Inflammatory Bowel Disease (IBD) as a whole and the risk of developing Breast Cancer (BC), with this overall positive correlation possibly attributed to its subtype, Crohn’s Disease (CD), as further Mendelian Randomization analysis revealed a positive correlation between genetically predicted CD and BC risk. However, genetically predicted Ulcerative Colitis (UC) did not show a significant correlation with BC risk. Additionally, reverse-direction Mendelian Randomization analysis indicated a strong positive correlation between genetically predicted BC and the overall risk of IBD, but not with its subtypes CD and UC. Bidirectional two-sample Mendelian randomization analysis based on ER+ and ER-breast cancer subtypes indicates that ER-breast cancer increases the overall incidence of IBD, whereas ER + breast cancer does not show a positive tendency towards promoting IBD. This suggests that the increased risk of IBD associated with breast cancer may be driven by the ER-subtype. While Crohn’s disease (CD) raises the overall risk of breast cancer, there is insufficient evidence to prove that CD specifically increases the risk of ER-breast cancer. This implies that CD may elevate the overall breast cancer risk by increasing the risk of ER + breast cancer. These findings are consistent with the results of our study.

Current studies on the correlation between IBD and BC risk are limited and of low evidence level, unable to definitively confirm the causal relationship between IBD and its subtypes with BC risk, and *vice versa*. Can Gong and colleagues conducted a meta-analysis (Gong et al., 2022) incorporating 16 cohort studies, which collectively assessed the incidence of breast cancer following a diagnosis of inflammatory bowel disease (IBD). This analysis calculated the pooled odds ratio (OR) and 95% confidence intervals (CIs) to evaluate the relationship between IBD and the risk of breast cancer. The findings indicated that the overall risk of breast cancer among patients with IBD was not significantly increased, with a pooled OR of 0.94 (95% CI, 0.82–1.06). Subgroup analyses revealed that the ORs for patients with Crohn’s disease and ulcerative colitis were 0.91 (95% CI, 0.70–1.12) and 0.99 (95% CI, 0.90–1.08), respectively, neither of which showed a significant association. Geographical differences suggested a slightly higher OR in the Asian population at 1.01 (95% CI, 0.73–1.30) compared to the European population at 0.90 (95% CI, 0.75–1.06), although the difference was not substantial. Therefore, this study demonstrates that neither in Asian nor European populations does IBD, nor its subtypes Crohn’s disease (CD) and ulcerative colitis (UC), significantly influence the risk of developing breast cancer. This conclusion contradicts the findings of our study, although our results suggest a significant but not prominent positive correlation between IBD as a whole and BC risk, indicating a possible positive correlation between IBD and its subtypes with BC risk. This difference in conclusions could be related to the fewer number of studies included in the meta-analysis, with a lower proportion of European population patients. To date, there is still a lack of large cohort studies or randomized controlled trials to confirm the bidirectional causal relationship between IBD and BC. However, several Mendelian Randomization studies have already confirmed the association between IBD, intestinal tumors, and an increased risk of extraintestinal tumors. A meta-analysis (Gao et al., 2023) of a Mendelian randomization study exploring the causal links between inflammatory bowel disease (IBD) and 32 types of extracolonic cancers suggests a potential causal relationship between IBD and its subtypes—Crohn’s disease (CD) and ulcerative colitis (UC)—and oral cancer. Specifically, the odds ratio (OR) for IBD is 1.180 with a 95% confidence interval (CI) from 1.059 to 1.316, with a *p*-value of 0.003. For CD, the OR is 1.112, with a 95% CI from 1.008 to 1.227, and a *p*-value of 0.034; for UC, the OR is 1.158, with a 95% CI from 1.041 to 1.288, and a *p*-value of 0.007. Moreover, aggregated data from multiple Mendelian randomization studies reveal a significant positive causal relationship between IBD and breast cancer, with an OR of 1.059, 95% CI from 1.033 to 1.086, and a *p*-value less than 0.0001, and a potential causal connection between Crohn’s disease and breast cancer, indicated by an OR of 1.029, 95% CI from 1.002 to 1.055, with a *p*-value of 0.032. This meta-analysis clarifies the potential causal associations of IBD and its subtypes with the incidence of both oral and breast cancers. The results are consistent with our study, yielding similar ORs (IBD: OR = 1.059; 95% CI: 1.033–1.086; *p* < 0.0001 vs. OR = 1.047; 95% CI: 1.009–1.087; *p* = 0.014; CD: OR = 1.029; 95% CI: 1.002–1.055; *p* = 0.032 vs. OR = 1.044; 95% CI: 1.015–1.073; *p* = 0.002). Our study employs bidirectional Mendelian randomization, enhancing the robustness of these conclusions. Additionally, our research provides a theoretical basis for refined conclusions and future precision medicine by analyzing the associations between IBD and its subtypes with various breast cancer subtypes characterized by differing estrogen receptor expression statuses using bidirectional Mendelian randomization. Additionally, the study by Chengdong Yu et al. (Yu et al., 2023) also explored the causal relationship between Crohn’s disease and the incidence of breast cancer overall and its subtypes. The results indicated that genetically predicted Crohn’s disease could increase the risk of overall breast cancer, ER + breast cancer, and ER-breast cancer. However, after adjusting for smoking and alcohol consumption in the multivariable MR analysis, the positive contribution of Crohn’s disease to ER-breast cancer disappeared. This suggests that the contribution of Crohn’s disease to ER-breast cancer may be due to confounding factors related to concurrent smoking and/or alcohol consumption. The adjusted conclusions of their study align closely with the conclusions of our study, further confirming the robustness of our findings. A two-sample Mendelian Randomization study (Huang et al., 2023) by Jinsheng Huang et al. confirmed that IBD increased the incidence of pancreatic cancer by 1.22 times and hepatocellular carcinoma by 1.28 times, and also established a positive correlation between Ulcerative Colitis and an increased risk of cholangiocarcinoma. At the current stage of research, pro-inflammatory cytokine IL1β (Däbritz and Menheniott, 2014) is a key determinant of the risk of human inflammation-related cancers, capable of upregulating inducible nitric oxide levels, thereby abnormally regulating and increasing the activity of nitric oxide-dependent DNMT1 (Wong, 2021; Zhu et al., 2022) (DNA Methyltransferase 1), leading to abnormal silencing or expression of certain genes, especially as over-methylation in gene promoter regions can silence tumor suppressor genes, leading to tumorigenesis. This partially elucidates the molecular mechanisms underlying the association between inflammatory bowel disease (IBD) and cancer. Furthermore, the potential mechanistic link between the formation of breast cancer and IBD includes abnormalities in the immune environment within the breast and systemic inflammatory responses. These factors may lead to a reduction in the expression of breast cancer resistance protein (BCRP) and an increase in the expression of G protein-coupled estrogen receptor (GPER) on the membrane of breast cells. Subsequently, these alterations in biological pathways ultimately promote the development of breast cancer. Therefore, there is a plausible basis to consider a bidirectional causal relationship between IBD and breast cancer, potentially related to this molecular mechanism.

## Strengths and limitations

The strength of this study lies in its use of two-sample Mendelian randomization to investigate the bidirectional causal relationships between inflammatory bowel disease (IBD) and its subtypes (Crohn’s disease [CD] and ulcerative colitis [UC]) and the risk of breast cancer and its subtypes (ER + BC and ER-BC). Compared to observational studies, this approach simulates a randomized controlled trial, minimizing susceptibility to confounding factors and thus providing a higher level of evidence. Additionally, the study employed the Egger-intercept method for horizontal pleiotropy testing, which confirmed the reliability of the results.

However, there are limitations to this study. The Mendelian randomization analysis was conducted based on European populations, which may limit the generalizability of the findings across different ethnic groups. Furthermore, although the results of the forward Mendelian randomization study demonstrated a significant positive association between IBD, CD, and breast cancer, the odds ratios were relatively small. This indicates that while IBD and CD increase the risk of breast cancer, the extent of this increase is limited. Additionally, due to constraints in the composition of the GWAS database, this study only performed bidirectional two-sample Mendelian randomization analyses on ER+ and ER-breast cancer subtypes, precluding further analysis based on different HER2, PR, or even AR expression statuses. Consequently, the fine-tuned generalization and clinical application of the study’s conclusions remain limited.

## Conclusion

This study marks the beginning of research into the association between IBD and BC. It successfully bridges the interdisciplinary research between chronic inflammatory bowel diseases and extraintestinal tumors, and hints at the importance of preventive screening for populations of IBD and BC patients. The disease mechanism of IBD is complex, affecting a wide range of cancerous and non-cancerous diseases. Future research should further explore the molecular mechanisms and differential gene expression related to the occurrence of IBD and cancerous as well as non-cancerous diseases.

## Data Availability

The original contributions presented in the study are included in the article/Supplementary Material, further inquiries can be directed to the corresponding author.
