# Supplementary material for: The causal association between inflammatory bowel disease and breast cancer: a bidirectional two-sample Mendelian randomization study
Source: Front Genet. 2024 Jul 23;15:1392341. doi: 10.3389/fgene.2024.1392341 (PMC11300198; doi:10.3389/fgene.2024.1392341)
Supplement: Supplementary file 5 [file Table2.DOCX]

**The causal association between inflammatory bowel disease and breast cancer: A bidirectional two-sample Mendelian randomization study**

YIN Yulai^1^, ZHANG Xiaoyu^2*^

(1.Cangzhou Central Hospital, Hebei Medical University,Cangzhou 061000,China;2.Department of Thyroid and Breast Surgery Ⅲ, Cangzhou Central Hospital,Cangzhou 061000,China)

Corresponding author:

Name: Xiaoyu ZHANG

E-mail:17631723184@163.com
